# Functional Decoupling of Emotion Coping Network Subsides Automatic Emotion Regulation by Implementation Intention

**DOI:** 10.1155/2021/6639739

**Published:** 2021-01-05

**Authors:** Shengdong Chen, Nanxiang Ding, Fushun Wang, Zhihao Li, Shaozheng Qin, Bharat B. Biswal, Jiajin Yuan

**Affiliations:** ^1^The Laboratory for Affect Cognition and Regulation (ACRLAB), Institute of Brain and Psychological Sciences, Sichuan Normal University, Chengdu, China; ^2^School of Psychology, Southwest University, Chongqing, China; ^3^School of Psychology, Shenzhen University, Shenzhen, Guangdong, China; ^4^State Key Laboratory of Cognitive Neuroscience and Learning & IDG/McGovern Institute for Brain Research, Beijing Normal University, Beijing 100875, China; ^5^Department of Biomedical Engineering, New Jersey Institute of Technology, Newark, New Jersey, USA

## Abstract

Automatic emotion regulation (AER) plays a vital role in the neuropathology underlying both suicide and self-harm via modifying emotional impact effortlessly. However, both the effortless account and the neural mechanisms of AER are undetermined. To investigate the neural changes at AER, we collected functional MRI (fMRI) in 31 participants who attended to neutral and disgust pictures in three conditions: watching, goal intention (GI), and reappraisal by implementation intention (RII). Results showed that RII (but not GI) decreased negative feelings and bilateral amygdala activity without increasing cognitive efforts, evidenced by the reduced effort rating and less prefrontal engagement during RII compared with during watching and GI. These emotion-regulatory effects of RII cannot be explained by emotional habituation, as the supplementary experiment (*N* = 31) showed no emotional habituation effects when the same disgust pictures were presented repeatedly three times for each watching and GI condition. Task-based network analysis showed both RII and GI relative to watching increased functional connectivities (FCs) of the ventral anterior cingulate cortex to the left insula and right precuneus during conditions, two FCs subserving goal setup. However, RII relative to GI exhibited weaker FCs in brain networks subserving effortful control, memory retrieval, aversive anticipation, and motor planning. In these FCs, the FC intensity of putamen-operculum/lingual and paracentral-superior temporal gyri positively predicted regulatory difficulty ratings. These findings suggest that the setup of implementation intention automatizes emotion regulation by reducing the online mobilization of emotion-coping neural systems.

## 1. Introduction

Emotion regulation, a process to modulate any components of emotional activity [1], plays a vital role in maintaining one's health and avoiding suicide or self-harm. Though it has been suggested that emotion regulation can be realized by either effortful or automatic process [2], most studies to date focus on the effortful forms of emotion regulation that is resource demanding. For instance, neuroimaging studies found that intentional reappraisal decreased emotional experience and emotion-related subcortical activation (e.g., amygdala) at the cost of increasing control-related prefrontal activation [3, 4]. The cognitive cost, in some instances, may lead to a failure of emotion regulation. For example, individuals with major depressive disorder are characterized by deficits in prefrontal cognitive control function, which consequently leads to disinhibition of negative emotion [5].

An increasing number of behavioral and electrophysiological studies have recently examined automatic or implicit forms of emotion regulation [6–8]. The recent two-dimensional framework proposed that these different forms of emotion regulation can be organized along two orthogonal psychological dimensions: (a) the nature of emotion regulation goal, ranging from implicit/nonconscious to explicit/conscious and (b) the nature of the emotion change process, ranging from more automatic to more controlled [9]. Across these different forms of emotion regulation, implementation intention, most broadly, tends to produce some of the largest effect size (*d*_+_ = 0.91) relative to passive watching (control condition; [10]). More importantly, though the antecedent buildup of implementation intentions is cognitively demanding (i.e., conscious and explicit), the execution of emotion regulation process by implementation intentions is automatic, without involving intentional regulatory efforts upon emotional stimulation [11, 12].

Implementation intentions were proposed to promote the attainment rate of goal intentions [13]. Goal intention (GI) defines desired end states and has the general format of “I want to attain Z!” (e.g., “I will not get upset!”). However, people often struggle to regulate their emotional responses with only such a goal intention to regulate emotions [6, 14]. By specifying when, where, and how goal-directed behaviors should be initiated in the form of “if-then” plan (e.g., “If loss sign is encountered, then I will keep calm!”), implementation intention links a goal-relevant situational cue (e.g., loss sign) with a goal-directed behavior (e.g., “keep calm”), which reduces the intention–behavior gap between intended outcome and actual goal attainment [15]. Gallo and Gollwitzer [16] first reported that forming implementation intention for the control of spider phobia reduced the subjects' fearful experience for spider-related stimuli during a cognitive demanding task. Additionally, an event-related potential (ERP) study by Gallo et al. [11] reported that forming an implementation intention reduced occipital P1 amplitudes for threatening stimuli compared to GI or watching conditions. Recently, Gallo et al. [12] found that a reappraisal-based implementation intention (RII) allowed participants to rate disgusting pictures as being less unpleasant than participants in the watching or GI groups.

However, there were two limitations in these previous studies of implementation intention. First, these studies did not use objective indexes to measure cognitive costs between regulation and no-regulation conditions, which were unable to verify the effortless or automatic characteristics of implementation intention. Specifically, Gallo and colleagues [11, 12] only collected subjective measures of cognitive efforts during goal intention and implementation intention conditions, but not during the control condition (i.e., passive watching), leaving it unclear whether implementation intention or goal intention enhanced cognitive efforts compared to the control condition. Second, what these studies measured are self-reported or electrophysiological variables. Few studies have examined neural mechanisms of automatic emotion regulation by implementation intention using functional MRI (fMRI). The only exception was one fMRI study by Hallam et al. [17] that involved implementation intention and cognitive reappraisal. However, the RII was manipulated in a controlled (not automatic) way in this study, i.e., participants were reminded to use the RII strategy every time a reappraisal cue was presented. Though RII reduced self-reported affect and amygdala activity compared to goal intention, RII also led to cognitive control-related activation in dorsolateral prefrontal regions [17].

Therefore, we here performed one fMRI experiment to examine the automatic characteristic and neural mechanisms of negative emotion regulation by RII. In the fMRI experiment, cognitive reappraisal, which requires reformulating the meaning of the emotional situation, was chosen as the target strategy to be planted into implementation intention. Intentional cognitive reappraisal has been suggested to effectively reduce negative emotional outcomes, which, however, was commonly accompanied by increased cognitive efforts at both neural [3] and behavioral [18] levels. Disgust was chosen as the target emotion, as it has proven to elicit robust neural activation in both cognitive-control and emotion-generative regions (e.g., amygdala) [3, 19, 20].

Furthermore, the amygdala and insula have been suggested to be central regions underlying the generation of disgust [3, 19, 20]. Prefrontal regions like dorsolateral prefrontal cortex (dlPFC) and dorsal anterior cingulate cortex (dACC) are consistently involved in controlled reappraisal and play a critical role in top-down cognitive control [3, 4]. Therefore, neural activity in these two emotion-generative regions (amygdala and insula) and two cognitive control-related regions (dlPFC and dACC) were used to provide objective indexes of emotional responses and cognitive costs, respectively. We predicted that RII would reduce the subjective experience of disgust without increasing subjective reports of cognitive efforts. We also predicted that RII would reduce the disgust responses in emotion-generative regions without increasing the cognitive control regions' activity compared to GI or control condition (i.e., automatically). Furthermore, we conducted a voxel-wise whole-brain analysis and a task-related network analysis to explore the neural mechanisms subserving the automatic emotion regulation by RII.

## 2. Materials and Methods

### 2.1. Participants

Given our aim to compare the negative emotional responses among the two regulations (GI and RII) and one control (watching) condition, we determined the sample size based on a power analysis using the G-power software for repeated ANOVA [21]. We specified a medium effect size (0.25), 0.8 power, and a moderate correlation (0.5) among the repeated measurements (3), which yielded a recommended sample size of 28. We used a medium effect size (*f* = 0.25) [22] because the values found in previous studies (average *f* = 0.45) [12, 14] would yield a very small sample size (9). To avoid the possibility that some of the data cannot be used because of head movement or other uncontrollable reasons, we employed thirty-one healthy, right-handed college students (16 males, *M*_age_ = 21.34) from the Southwest University in China with the normal or corrected-to-normal vision to participant in this study. Written informed consent was obtained before the experiment, and the study was approved by the local ethical committee of the Institutional Human Participants Review Board of the Southwest University Imaging Center. Data of 5 participants were excluded due to excessive head movement (larger than 3 mm) during fMRI scanning.

### 2.2. Stimuli

The stimulus material consisted of two categories with 90 pictures in total: 45 disgust and 45 neutral pictures, taken from the International Affective Picture System (IAPS) [23] and the Chinese Affective Picture System (CAPS) [24]. Each picture's valence and arousal scores were assessed by 30 independent raters, independent of the experiment sample. The disgust pictures showed bloody burn victims and mutilated bodies. Within the bidimensional valence and arousal model, such contents are rated as negative and high arousal, while neutral pictures had medium standard emotional valence and low arousal ratings. The pictures were presented in a randomized order, and the raters blind to the experiment intention were asked to rate to what degree they felt sadness, fear, joy, disgust, and anger on scales ranging from 1 (little) to 7 (very). Results revealed a significant main effect for the unpleasant pictures, (*F*(4, 40) = 1088.96, *p* < 0.001, and *η*^2^ = 0.96). Post hoc Bonferroni tests showed that disgust pictures were rated as being more likely to produce disgust (*M* = 5.74) when compared with fear (*M* = 4.22, *p* < 0.001), sadness (*M* = 3.90, *p* < 0.001), anger (*M* = 2.85, *p* < 0.001), and joy (*M* = 1.41, *p* < 0.001). These findings suggest that unpleasant pictures elicit disgust effectively.

### 2.3. Design and Procedure

The present study used a three × two factorial design with the regulation condition (watching, GI, and RII) and type of pictures (neutral/negative) as two repeated factors.

Prior to fMRI scanning, participants were trained to be familiar with the experimental task while viewing 15 practice pictures. Participants were told to estimate their negative emotional intensity after the presentation of 3 consecutive pictures using a five-point scale ranging from 0 (not at all) to 4 (very), “How negative did you feel?” After viewing all the pictures, participants received a questionnaire that assessed the consumption of cognitive resources: “How much did you try to cope with negative feelings?” and “How difficult was it to cope with negative feelings?” The two items were also accompanied by a five-point scale ranging from 0 (not at all) to 4 (very). The individual difference in the habitual use of reappraisal was measured before fMRI scanning using the Emotion Regulation Questionnaire (ERQ; [25]).

**Figure 1:**
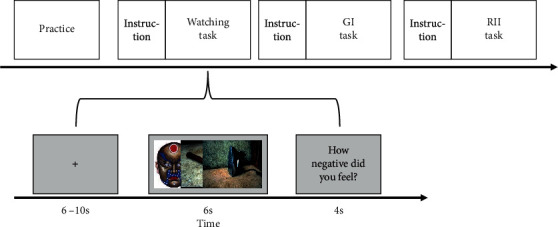
Experimental design for three tasks. The experiment consisted of three tasks, and each task consisted of five neutral and five negative blocks. “How negative did you feel?” rating (0 = not at all to 2 = moderately to 4 = extremely) appeared at the end of each block. GI: goal intention; RII: reappraisal-based implementation intention.

During fMRI, participants performed three tasks in turn: passive watching, GI, and RII (Figure 1). When performing each of the three tasks, participants first received the task instruction and reinforced it by rehearsal for one minute. Specifically, participants in the watching task were just required to pay close attention to the pictures without further instructions related to emotion regulation. Besides paying close attention to pictures, participants in the GI task were instructed to form a goal intention (“I will not get disgusted!”). In the RII task, participants were instructed first to form a goal intention and a goal-directed if-then plan (“I will not get disgusted! And if I see blood, then I will take the perspective of a physician!”).

After the instruction stage, participants in each task only needed to attend to the pictures without any additional cues or instruction to avoid any further voluntary regulatory process. Each task consisted of 10 blocks (5 neutral and 5 negative blocks) that matched in valence and arousal, and each block consisted of 3 consecutive pictures (2 s each) of the same valence. Both neutral and negative pictures across the three tasks were not significantly different in valence and arousal (*p*s > 0.8). Each of the 10 blocks was presented for 6 s in a random order following a fixation of 6 to 10 s (average 8 s). Following each block, the scale to assess negative emotion intensity appeared on the screen for 4 s. At the end of each task, two scales to assess cognitive efforts in emotional coping were also presented for 4 s each. There was no missing data on any of the subjective measures.

### 2.4. Imaging Data Acquisition and Preprocessing

Data were acquired on a Siemens Trio 3.0 Tesla (Magnetom Trio, Siemens, Erlangen, Germany) scanner with a gradient echo planar imaging sequence (32 axial slices, TR/TE = 2 s/30 ms, FA = 900, matrix size = 64 × 64, FOV = 220 × 220 mm^2^, voxel size = 3.4 × 3.4 × 3 mm^3^, and 386 volume measures). High-resolution structural images were acquired for registration purposes using a T1-weighted magnetization-prepared rapid gradient-echo (MP-RAGE) sequence (TR/TE = 1900 ms/2.52 ms, FA = 9°, FOV = 256 × 256 mm^2^, slices = 176, thickness = 1.0 mm, and voxel size = 1 × 1 × 1 mm^3^). SPM8 [26] was used for the fMRI data analysis with regular preprocessing steps of slice timing, realignment, volume registration, spatial normalization (resampled into 3 mm isotropic voxels), and spatial smoothing with a Gaussian kernel of 8 mm full width at half maximum. Head movement estimates derived from the realignment step were included as nuisance regressors in subsequent general linear modeling (GLM) to diminish the impact of movement-related effects.

### 2.5. Imaging Data Analysis

For each participant, the voxel-wise whole-brain GLM included 6 regressors of interest (negative and neutral pictures in three scans of watching, GI, and RII). At the group level, a general linear contrast of watching-negative versus watching-neutral was applied to detect brain activation associated with disgust responses. Based on Random Field Theory, *T*-statistics for each voxel were thresholded at *p* < 0.01 and an extent of 10 voxels for multiple comparisons across the whole brain with a family-wise error rate (FWE).

Region of interest (ROI) analyses were next performed to test whether RII could decrease activation related to disgust responses in typical emotion-generative regions. The emotion-generative regions (bilateral amygdala and bilateral insula) were defined by the respective anatomical masks of the AAL atlas [27] by WFU_PickAtlas toolbox [28]. To examine whether RII increased activation in cognitive control-related regions, three ROIs in bilateral dlPFC and dACC were further defined as the Brodmann areas 9 and 46 combined (left and right), as well as a 10 mm radius sphere at Talairach coordinates (*X* = 0, *Y* = 12, and *Z* = 42) [29], respectively. For each of these ROIs, the mean percent signal changes (PSCs) of each individual were extracted using the Marsbar toolbox [30]. The emotional intensity of negative affect during each condition was represented as the average contrast values (negative > neutral) for the four emotion-generative regions and three cognitive control-related regions.

### 2.6. Estimation of Task-Related FC

Using the CONN toolbox [31], we estimated the task-related FC between each pair of brain regions in a network of 229 spherical (radius = 3 mm) regions. Of these regions, 227 ROIs were selected from 264 coordinates reported by Power et al. [32]. Those coordinates are the centers of putative functional areas (and subcortical and cerebellar nuclei), which were defined by multiple-task fMRI meta-analyses [33] and by a resting-state FC MRI parcellation technique [34]. These 227 ROIs have also been assigned to 10 well-established functional networks, comprising low-level input and output networks (visual, auditory, and sensorimotor networks), subcortical nodes, the default mode network (DMN), ventral and dorsal attention networks (VAN and DAN), and cognitive control networks (frontal-parietal network, FPN; cingulo-opercular network, CON; salience network, SN; [35, 36]). Bilateral amygdala ROIs defined via the contrast of watching − negative > watching − neutral (S-Table 1) were also added in the connectivity analysis given their central role in the processing of disgust.

Regional time series within each of these 229 ROIs were extracted from the preprocessed fMRI data on individual level. The task onset times were modeled, and covariates of no interest (e.g., the realignment confound, white matter, and CSF signal) were regressed out using a component-based noise correction method (CompCor) [37]. Time series of voxels within 229 ROIs were averaged, and those average time series were correlated with each other. The resulting correlation coefficients were then Fisher z-transformed to normalize their distribution. These values represent the connectivity between the source and target regions during each task condition. The computed ROI-to-ROI connectivity matrices of each participant were finally entered into the second-level group analysis that treated participants as a random variable in a 3-by-2 ANOVA. In this network analysis, false positives were controlled by the false discovery rate (FDR) of *p* < 0.05.

### 2.7. Statistical Analysis

We conducted the null hypothesis significance test (NHST) analysis using SPSS 20.0. We also conducted Bayesian ANOVA to analyze cognitive efforts' subjective and neural measures, mainly because we expected that RII would not increase cognitive efforts relative to the watching condition. We computed Bayes factors (BF_10_) using JASP with default prior width [38]. We interpreted BF_10_ of 1-3 as anecdotal, 3–10 as moderate, >10 as strong evidence for accepting H1, and BF_10_ of 1-1/3 as anecdotal, 1/3–1/10 as moderate, and <1/10 as strong evidence for accepting H0 [39].

## 3. Results

### 3.1. Manipulation Check of Emotion Induction

To check whether the disgusting pictures elicited the target emotion disgust, we contrasted watching-negative versus watching-neutral on both experience and neural measures of negative emotion during the passive watching. Results showed that the watching-negative versus watching-neutral contrast resulted in increased subjective ratings of negative affect (*t*(25) = 28.49 and *p* < 0.01) (S-Figure 1A) and increased responses in prefrontal cortex, bilateral temporal and occipital cortex, parietal cortex, and subcortical regions (S-Table 1). As expected, we confirmed that the typical emotion-generative regions, including the amygdala and insula, were bilaterally activated by this contrast (S-Figure 1B). There were no significant brain responses for watching-neutral versus watching-negative contrast. Both behavioral and neural findings supported that the disgusting pictures successfully elicited the target emotion of disgust.

### 3.2. Subjective and Neural Emotion Regulation Effects

The univariate ANOVAs of negative emotional ratings and the percent BOLD signal changes in the key emotion-generative regions (amygdala and insula) were conducted among the three conditions (watching, GI, and RII). At both the behavioral and neural levels, the intensity of negative affect during each condition was represented by the negative emotion index minus the neutral emotion index. Its higher values mean more negatively emotional intensity during the condition.

#### 3.2.1. Negative Emotional Ratings

**Figure 2:**
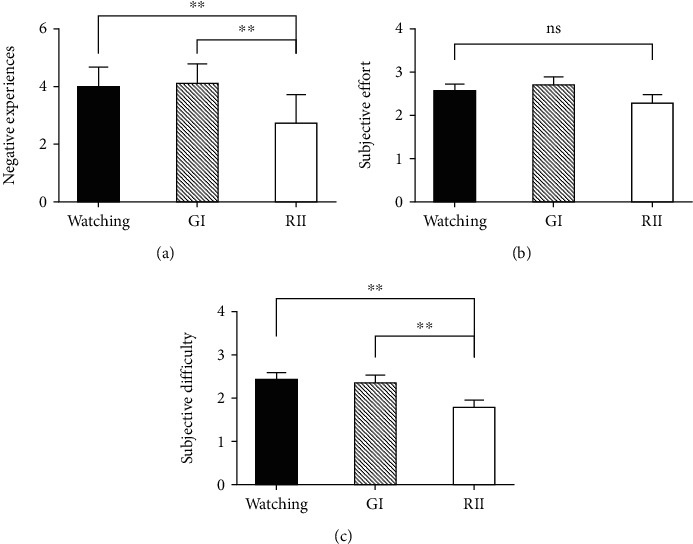
The subjective negative experience (a), subjective effort (b), and subjective difficulty (c) ratings of the watching, GI, and RII conditions. Error bars = SEM; ^∗∗^ means *p* < 0.01; ns stands for not significant.

There were significant differences in negative emotional ratings across the three conditions (Figure 2(a)), at both before (*F*(2, 50) = 29.82, *p* < 0.001, and *η*^2^ = 0.54) and after (*F*(2, 49) = 6.62, *p* < 0.01, and *η*^2^ = 0.22) taking habitual reappraisal as a covariate. Bonferroni post hoc comparisons indicated that the RII condition (*M* = 2.74) had a significantly lower negative emotional level than the watching condition (*M* = 3.98, *p* < 0.001) and the GI condition (*M* = 4.11, *p* < 0.001). The watching condition and the GI condition showed no significant differences in negative emotion experience, (*p* = 1.0).

#### 3.2.2. Neural Responses in Emotion-Generative ROIs

**Figure 3:**
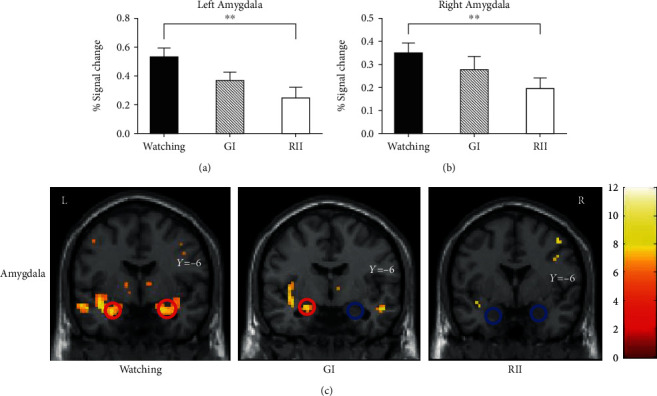
The emotion-regulatory effects of RII in the (a) left and (b) right amygdala ROIs. The intensity of negative affect during each condition was represented by the BOLD signal changes of the negative block versus the neutral block. (c) An illustration of amygdala activation among watching, GI, and RII conditions (FWE corrected *p* = .01 and an extent of 10 voxels). Error bars = SEM; ^∗∗^ means *p* < 0.01.

There were significant main effects in bilateral amygdala responses (left/right amygdala: *F*(2, 50) = 6.43/3.62, *p* = 0.003/0.03, and *η*^2^ = 0.20/0.13; Figures 3(a)–3(c)). In Bonferroni post hoc comparisons that in the left amygdala, PSC (*M* = 0.25) was smaller during RII than in watching conditions (*M* = 0.53, *p* = 0.008). The watching and the GI (0.37) conditions showed no significant differences (*p* = 0.10). Similarly, in the right amygdala, PSC during the RII condition (*M* = 0.20) was also smaller than the watching condition (*M* = 0.36, *p* = 0.02). The watching condition and the GI (*M* = 0.28) condition showed no significant differences (*p* = 0.68). However, no significant main effects were found in the left insula (*F*(2, 50) = 0.90, *p* = 0.42, and *η*^2^ = 0.04) or in the right insula responses (*F*(2, 50) = 0.51, *p* = 0.60, and *η*^2^ = 0.02).

These findings showed that forming implementation intention effectively realized emotion-regulatory goals, reducing negative feelings and disgust-related neural activations (bilateral amygdala).

### 3.3. Subjective and Neural Cognitive Costs of Reappraisal-Based Implementation Intention

We conducted the univariate ANOVA analysis of both subjective cognitive efforts and the percent BOLD signal changes in the bilateral dlPFC and dACC among the three conditions (watching, GI, and RII).

#### 3.3.1. Subjective Cognitive Efforts

No significant differences in the subjective effort of negative emotion regulation emerged among the watching (*M* = 2.57), the GI (*M* = 2.69), and the implementation intention conditions (*M* = 2.27, Figure 2(b)); *F*(2, 50) = 1.82; *p* = 0.17; BF_10_ = 0.487. The self-reported difficulties in coping with negative emotions were significantly different among the watching (*M* = 2.42), GI (*M* = 2.35), and RII conditions (*M* = 1.73, Figure 2(c)); *F*(2, 50) = 7.64; *p* < 0.01; *η*^2^ = 0.23; BF_10_ = 34.08. The RII condition was linked with a significantly lower report of difficulties than the watching (*F*(1, 25) = 14.46, *p* < 0.01, *η*^2^ = 0.37, and BF_10_ = 40.95) and GI conditions (*F*(1, 25) = 7.66, *p* = 0.01, *η*^2^ = 0.23, and BF_10_ = 4.53), whereas GI and watching showed no significant differences (*F*(1, 25) = 0.19, *p* = 0.66, and BF_10_ = 0.226).

#### 3.3.2. Neural Responses in Cognitive Control-Related ROIs

**Figure 4:**
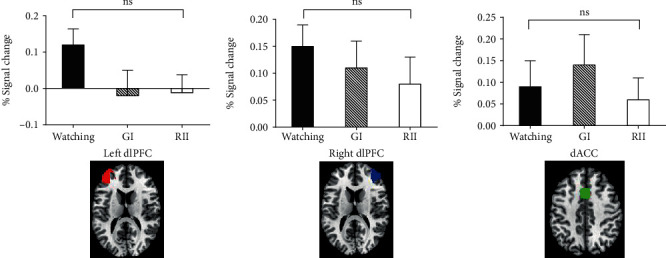
The neural responses of the left and right dlPFC and dACC in the watching, goal intention (GI), and reappraisal by implementation intention (RII) conditions. Error bars = SEM; ns stands for not significant.

To check whether implementation intention engenders voluntary control in the neural level, we directly tested the BOLD signal changes of key nodes of the frontoparietal control network. The main effects of BOLD responses in the right (BF_10_ = 0.196) and left (BF_10_ = 0.773) dlPFC and dACC (BF_10_ = 0.705) ROIs were not significant (*p*s > 0.10) (Figure 4).

#### 3.3.3. Whole-Brain Analyses

**Figure 5:**
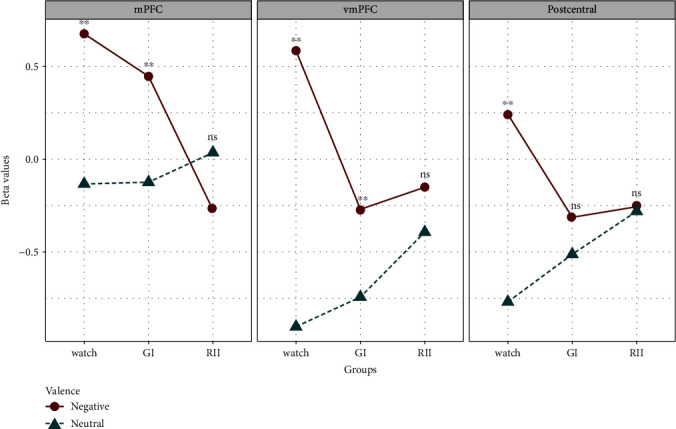
Changes of beta values of activation clusters for the interaction effects (picture type ^∗^ strategy type). ^∗∗^ means *p* < 0.01; ns stands for not significant.

Moreover, to test whether, in addition to the regions of interest, other regions were affected by RII, a 3-by-2 repeated-measure ANOVA was run in a whole-brain analysis with picture type and strategy type as factors. The strongest interaction effect was found in the left mPFC, vmPFC, and postcentral regions (Table 1). Follow-up *t*-tests showed that the difference between negative and neutral block was significant during both GI (left mPFC: *t* = 4.63, *p* < 0.001; vmPFC: *t* = 2.58, *p* < 0.02) and watching conditions (left mPFC: *t* = 6.05, *p* < 0.001; vmPFC: *t* = 8.85, *p* < 0.001), but not during RII (left mPFC: *t* = −1.86, *p* = 0.075; vmPFC: *t* = 1.18, *p* = 0.25) in left mPFC and vmPFC. The difference between negative and neutral block at the postcentral region was only significant during the watching condition (*t* = 7.22, *p* < 0.001) but not during GI (*t* = 1.28, *p* = 0.21) and RII (*t* = 0.14, *p* = 0.89) (Figure 5). These findings indicate that RII did not increase disgust-related neural processing in the prefrontal regions.

Together, these behavioral and neuroimaging findings showed that RII facilitated downregulation of disgust responses without more cognitive resource costs compared to watching and GI conditions.

#### 3.3.4. Task-Related FC Analyses

We applied task-related FC analysis to the 3-by-2 experimental datasets by considering ROIs as nodes and the block-by-block FC between each pair of ROIs as edge intensity. After computing the ROIs pair-wise correlation matrix of each condition for each participant, we conducted a 3-by-2 repeated-measure ANOVA of FC with picture valence and type of strategies as factors at the group level.

**Figure 6:**
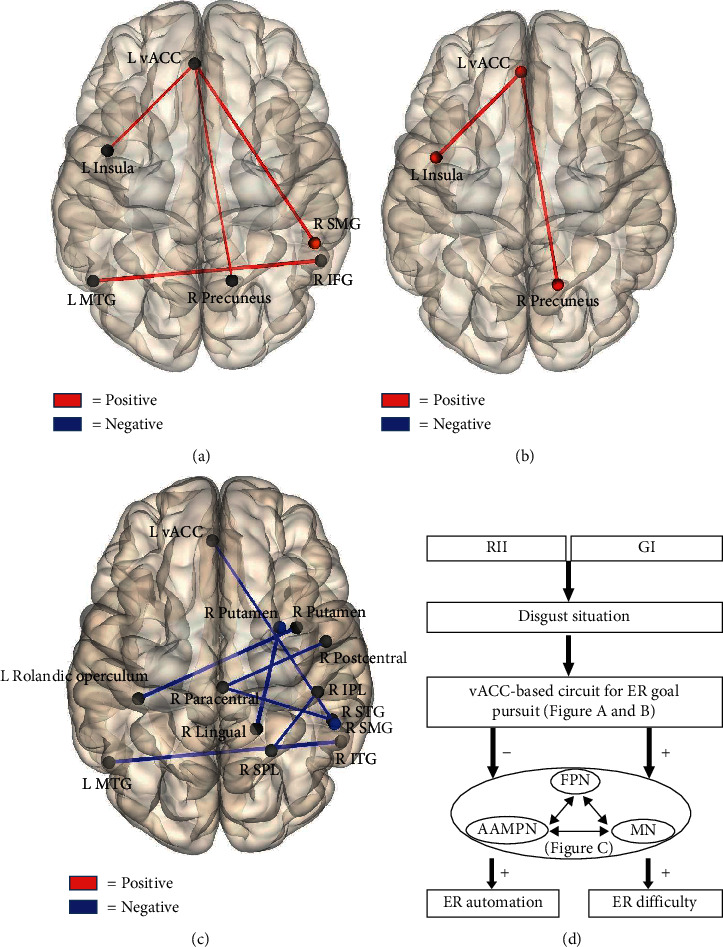
Functional connectivity patterns of the contrasts GI (a) and RII (b) versus watching condition and the contrast RII versus GI (c). The connections (edges) between ROIs marked in red mean that GI or RII relative to watching shows greater FC strength, and those marked in blue mean that RII relative to GI shows weaker FC intensity. ITG: inferior temporal gyri; MTG: middle temporal gyri; SMG: supramarginal gyri; STG: superior temporal gyri; IPL: inferior parietal lobule; SPL: superior parietal lobule; SMG: supramarginal gyri. (d) The dynamic architecture of the FC mechanisms underlying GI and RII. FPN: frontoparietal network; AAMPN: aversive anticipation and motor planning network; MN: memory network; ER: emotion regulation.

The strongest interaction effect was found in 12 pairs of ROI-to-ROI FC (corrected for multiple comparisons via FDR) (see S-Table 2). Planned comparisons for each FC were then conducted by testing how the FC intensity difference between negative and neutral blocks varies across the regulation conditions. Specifically, we were mainly interested in the contrasts GI/RII versus watching and RII versus GI. According to cognitive subtraction principle, the contrasts GI and RII versus watching condition should reflect the FC underlying the intentional and automatic pursuit of emotion regulation goals (GI and RII), respectively. The contrast RII versus GI should reflect FC related to the differences of automation between the goal-directed GI and stimulus-driven RII. The results of contrasts GI and RII versus watching condition showed close similarities: four significant FCs for the contrast GI versus watching condition and two of these four FCs for the contrast RII versus watching condition (see Table 2 and Figures 6(a) and 6(b)). Further, seven FCs showed significant decreases in FC intensity during RII than GI (see Table 2 and Figure 6(c)). These seven FCs, as discussed later, may constitute an interactive neural system underpinning online emotion-related coping. Accordingly, we guess the reduced functional coupling in this system might reflect less mobilization of online processing resources for the attainment of emotion regulatory goal during RII.

Moreover, we conducted a correlation analysis between the FC intensity and the subjective difficulty index during RII relative to GI to investigate whether the survived FC could predict behavioral markers of cognitive efforts. Both FC and subjective indexes of regulatory difficulty were computed by using GI minus RII. We focused on the subjective difficulty index because it was related to negative experiences (*r* = 0.39 and *p* = 0.024) during GI relative to RII after a correction of FDR 0.05 (S-Figure 2), whereas the cognitive effort index was not (*r* = 0.33 and *p* = 0.049). The correlation analysis demonstrated that three of seven FC intensity were positively correlated with subjective difficulty: R putamen-L Rolandic operculum, *r* = 0.54 and *p* = 0.002; R lingual gyri-R putamen, *r* = 0.35 and *p* = 0.04; R paracentral lobule-R STG, *r* = 0.41 and *p* = 0.02 (S-Figure 2BCD). However, only the correlation of R putamen-L Rolandic operculum survived an FDR of 0.05 correction for multiple comparisons.

## 4. Discussions

The present study examined the automatic emotion-regulatory effects of RII at both behavioral and neural levels and its underlying functional connectivity mechanisms. Consistent with previous studies [6, 12, 14], our results revealed that RII effectively decreased both negative experiences and brain activity in emotion-generative regions. Importantly, these emotion-regulatory effects were not achieved at the cost of greater involvement of cognitive control resources, as the RII did not increase self-reported efforts and control-related prefrontal activations. Furthermore, FC analysis results demonstrated close similarities between the contrast GI versus watching condition and the contrast RII versus watching condition in vACC-based FCs, and the connectivity intensity was decreased during RII than GI in seven distributed FCs.

We found that the RII effectively reduced the emotional experiences and activation of the bilateral amygdala relative to the passive watching condition. In contrast, the GI and watching conditions showed no significant differences at both behavioral and neural indices. These findings coincided well with previous findings that forming an implementation intention effectively downregulated the subjective experience of negative emotions [12, 16], amygdala activation [17], and occipital P1 event-related potential amplitudes [11]. These findings confirmed the effectiveness of RII in reducing negative emotional outcomes at both the behavioral and neural levels. Importantly, it should be noted that the emotion-regulatory effects of RII cannot be explained by emotional habituation. In the supplementary experiment (*N* = 31), we observed no habituation effects when repeating to present disgust pictures (S-Figure 3).

Importantly, we observed no enhancement of subjective efforts but reduced control difficulty during RII compared with those under the other two conditions. There was also no activity increase in the cognitive control-related ROIs (bilateral dlPFC and dACC) during RII relative to watching and GI. The dlPFC and dACC have been suggested to be generally involved in cognitive-resource-demanding tasks, such as working memory [40, 41], decision-making [42], and voluntary emotion regulation [3]. The increased activation of these regions is considered to represent increased cognitive control [3, 41]. Moreover, the whole-brain analysis showed that RII, relative to the watching and GI conditions, did not increase the emotion-related activity of mPFC and vmPFC. The mPFC and vmPFC play essential roles in the appraisal, expression, and regulation of negative emotion, similar to the cognitive control functions of dlPFC and dlPFC described above [43, 44]. These findings consistently suggest that emotion regulation by RII did not involve cognitive control mechanisms and operated automatically.

Moreover, FC analysis showed close similarities between the contrast GI versus watching condition and the contrast RII versus watching condition. Specifically, the FC intensity between left vACC and two nodes (right precuneus and left insula) was increased during both GI and RII than watching conditions. Given that RII builds upon the GI and the context-response association, it is reasonable to infer that these two FCs may be necessary for self-related emotion-regulatory goal pursuit, regardless of the degree of task automation. The left insula has been suggested to be a key node of SN [32], critical for developing and updating motivational states with specific associated actions (i.e., goals) [45]. And the vACC and precuneus are hubs of DMN, involved in self-relevant information processing [32]. Given the close association between SN and DMN [46], these two networks may interact to mark the emotionally salient stimuli and then to process it directed by the self-relevant goals (“I will not get disgusted”; Figure 6(d)).

Beyond the similarities between GI and RII, the connectivity intensity was decreased during RII than GI in seven distributed FCs. Previous studies have pointed out that GI is a goal-directed process, whereas RII is a stimulus-driven one [10, 13]. Therefore, we guess the increased FC intensity during GI compared to RII may reflect a goal-directed (top-down) online emotion-related coping that underlies the gap between emotion-regulatory goals and emotion-regulatory success. These seven FCs can be summarized into three networks that may cooperate to perform this process. First, the connection IPL-SPL, as a part of FPN, is involved in preparing and applying goal-directed (top-down) selection for stimuli and responses [47], and its activity increases with higher cognitive demand [48]. Second, the putamen-Rolandic operculum, vACC-SMG, postcentral-paracentral lobule, and paracentral lobule-STG connections are involved in aversive anticipation (postcentral-paracentral lobule and paracentral-STG) and emotion-related motor planning and preparation (putamen-Rolandic operculum and vACC-SMG). Specifically, anticipatory activity in the right postcentral gyrus and STG is associated with greater emotional responses and decreased regulation success [49]. Neural patterns of the postcentral gyrus and paracentral lobule are closely related to the averseness level [50]. Further, SMG is involved in planning goal-oriented actions [51]. Putamen and Rolandic operculum also play similar roles in motor planning [52] and execution [53]. Last, ITG, MTG, and lingual gyri are key nodes of memory systems, mediating interconnected memory functions, like establishing representations in long-term memory [54] and memory retrieval [55].

Together, these three networks may constitute a neural system subserving the goal-directed, online emotion coping mechanism, including components of cognitive control, memory reference, and retrieval, as well as aversive anticipation and motor planning. Without the antecedent formation of a situation-response association (e.g., if-then plan), participants may have mobilized this system upon receipt of stimulus to achieve their emotion-regulatory goals, leading to greater experienced regulatory difficulty during GI than RII. We also observed positive correlations between the FC (of R putamen-L Rolandic operculum and paracentral lobule-STG) intensity and self-rating of emotion coping difficulty. These correlations suggest that increased difficulty of emotion coping is coupled with the higher online mobilization of the emotion-related coping network, which provides a possible explanation for the little emotion regulation effect during GI. On the other hand, given that task automatization is accompanied by decreasing activation of the putamen [56] and FPN [36], the decreased FC intensity during the RII may reflect a greater degree of goal-dependent automaticity.

Several limitations need to be acknowledged. First, only healthy participants were studied, and it is therefore unclear whether our results are generalizable to clinical samples. Given the cognitive control deficits in individuals with emotional disorders (e.g., anxiety and depression), implementation intention, as a way of automatic emotion regulation, may facilitate the clinical population compared to voluntary strategies. Second, this study only combined implementation intention with cognitive reappraisal. However, there are other emotion regulation strategies, like attentional deployment or expressive suppression. The neural mechanisms underlying different emotion regulation strategies by implementation intentions may be different. Third, this study focused on the downregulation of negative emotional outcomes without measuring neural substrates underlying the GI and RII formation. It is possible that participants paid cognitive resources during the formation of implementation intentions before stimulus presentation. Thus, future studies need to assess both the formation and application of implementation intention concerning control-related neural underpinnings.

In summary, we found that RII effectively decreased negative emotional responses at both behavioral and neural levels without enhancing cognitive control resource involvement. Functional decoupling of emotion coping network may subserve automatic emotion regulation by implementation intention.

## Figures and Tables

**Table 1:** Voxel-wise group activations by 3-by-2 ANOVA with picture type and strategy type as repeated factors.

Brain regions	Brodmann	*X*	*Y*	*Z*	Voxels	*F* value
L mPFC	10	-6	51	9	104	21.78
	10	-9	54	27	23	17.79
L vmPFC	11	-12	45	-15	91	20.05
	11	-6	36	-18	16	19.58
L postcentral	6	-27	-33	66	13	18.77

Note: all clusters reached a significance level of *p* = 0.05 (FWE corrected). For each cluster, *X*, *Y*, and *Z*, MNI coordinates; L: left.

**Table 2:** Planned comparisons of functional connectivity intensity between watching, GI, and RII conditions.

FC	*t* value
GI vs. watching	
L vACC-R precuneus	4.78
L vACC-R SMG	4.77
L vACC-L insula	4.67
R ITG-L MTG	2.86
RII vs. watching	
L vACC-L insula	2.82
L vACC-R precuneus	2.53
RII vs. GI	
R lingual gyri-R putamen	-6.22
R ITG-L MTG	-6.13
R paracentral lobule-R STG	-4.66
R putamen-L Rolandic operculum	-5.98
R postcentral gyri-R paracentral lobule	-5.01
R IPL-R SPL	-4.70
L vACC-R SMG	-4.49

Note: all connections reached a significance level of two-tailed *p* < 0.05. FC: functional connectivity. For each connection, L: left; R: right. ITG: inferior temporal gyri; MTG: middle temporal gyri; STG: superior temporal gyri; IPL: inferior parietal lobule; SPL: superior parietal lobule; SMG: supramarginal gyri.

## Data Availability

All the data are available from the corresponding author on reasonable request.

## Supplementary Materials

Supplementary Materials S-Figure 1: the contrast of watching-negative versus watching-neutral on behavioral and neural indices. (a) Negative emotional ratings during passively viewing of disgust pictures (watching condition). Error bars = SEM. (b) From the one-sample *t*-test across all 26 participants for the contrast watching-negative versus watching-neutral. The display threshold was *p* = .01, FWE corrected and an extent of 10 voxels. ^∗∗^*p* ≤ 0.01, S-Figure 2: the correlation analysis showed that during GI relative to RII, subjective regulatory difficulty was related to greater (a) subjective cognitive efforts and (b) negative experiences, and that the changes of FC intensity were related to greater subjective regulatory difficulty. Results of (c) R putamen-L Rolandic operculum survived a FDR of 0.05 correction for multiple comparisons, whereas (d) R lingual gyri-R putamen and (e) R paracentral lobule-R STG did not. Contour line density in (a) and (b**)** means the extent of point overlap. S-Figure 3: mean subjective ratings of negative emotions for the watching and GI groups during three times. The negative affect during each condition was represented by the negative emotion rating minus the neutral emotion rating, and its higher values mean more negatively emotional experiences during the condition. Error bars = SEM; ns stands for not significant. S-Table 1: group activations for contrast watching-negative versus watching-neutral. S-Table 2: results of 3-by-2 ANOVA of functional connectivity analysis.
