# Supplementary material for: Functional Decoupling of Emotion Coping Network Subsides Automatic Emotion Regulation by Implementation Intention
Source: Neural Plast. 2021 Jan 5;2021:6639739. doi: 10.1155/2021/6639739 (PMC7803421; doi:10.1155/2021/6639739)
Supplement: Supplementary Materials — S-Figure 1: the contrast of watching-negative versus watching-neutral on behavioral and neural indices. (a) Negative emotional ratings during passively viewing of disgust pictures (watching condition). Error bars = SEM. (b) From the one-sample t-test across all 26 participants for the contrast watching-negative versus watching-neutral. The display threshold was p = .01, FWE corrected and an extent of 10 voxels. ∗∗p ≤ 0.01, S-Figure 2: the correlation analysis showed that during GI relative to RII, subjective regulatory difficulty was related to greater (a) subjective cognitive efforts and (b) negative experiences, and that the changes of FC intensity were related to greater subjective regulatory difficulty. Results of (c) R putamen-L Rolandic operculum survived a FDR of 0.05 correction for multiple comparisons, whereas (d) R lingual gyri-R putamen and (e) R paracentral lobule-R STG did not. Contour line density in (a) and (b) means the extent of point overlap. S-Figure 3: mean subjective ratings of negative emotions for the watching and GI groups during three times. The negative affect during each condition was represented by the negative emotion rating minus the neutral emotion rating, and its higher values mean more negatively emotional experiences during the condition. Error bars = SEM; ns stands for not significant. S-Table 1: group activations for contrast watching-negative versus watching-neutral. S-Table 2: results of 3-by-2 ANOVA of functional connectivity analysis. [file 6639739.f1.docx]

**SUPPLEMENTARY MATERIALS**

**Functional Decoupling of Emotion Coping Network Subsides Automatic Emotion Regulation by Implementation Intention**

Shengdong **Chen^1*^**, Nanxiang **Ding^2*^,** Fushun **Wang**^1^**^*^,** Zhihao **Li**^3^**,** Shaozheng **Qin**^4^**,** Jiajin **Yuan^1^**, **and** Bharat **Biswal**^5^

**^1^**The Laboratory for Affect Cognition and Regulation (ACRLAB), Institute of Brain and Psychological Sciences, Sichuan Normal University, Chengdu, China; ^2^School of Psychology, Southwest University, Chongqing, China; ^3^School of Psychology, Shenzhen University, Shenzhen, Guangdong, China; ^4^State Key Laboratory of Cognitive Neuroscience and Learning & IDG/McGovern Institute for Brain Research, Beijing Normal University, Beijing 100875, China; ^5^Department of Biomedical Engineering, New Jersey Institute of Technology, Newark, New Jersey, USA.

^*^These authors contributed equally to this work.

**Correspondence** should be addressed to: Prof. Jiajin Yuan, Institute of Brain and Psychological Sciences, Sichuan Normal University, Chengdu; E-mail address: yuanjiajin168@126.com; or yuanjiajin168@sicnu.edu.cn (J. Yuan)

**Method of Supplementary Experiment**

**Participants**

Forty healthy right-handed females completed the study (mean age=20.0), and were randomly divided into watching (n=20) and GI (n=20) groups. All participants had normal or corrected-to-normal vision, reported no history of neurological or psychiatric disorders. Trait anxiety, state anxiety and depression assessment did not differ between the experimental groups, as shown by their scores in the State (STA; t(38)=-1.26, p=0.22) and Trait (TAI; t(38)=0.94, p=0.35) Anxiety Inventory (STAI) (Spielberger, 1970) and Beck Depression Inventory-II (BDI-II) (Beck, Steer, & Brown, 1996) (BDI, t=0.67, p=0.51). Before admission to the study, all participants gave their written informed consent. This experiment was approved by the Institutional Review Board of the Southwest University and was in accordance with the latest revision of the Declaration of Helsinki.

**Stimuli**

The neutral and disgust pictures used in this experiment were identical to those used in the main experiment.

**Procedure**

The general procedure was identical to the procedure of main experiment except for the following changes: 1) the Self-Assessment Manikin (SAM) (Bradley & Lang, 1994) were used to assess the valence ratings with respect to each of the block presented, and the valence rating ranged from 1 (very happy) to 9 (very unhappy); 2) the watching or GI tasks needs to be performed for three times.

**Results of Supplementary Experiment**

Because the three emotion regulation conditions (watching, GI and RII) are presented in subsequent runs, potential emotional habituation or repeated formation of GI may influence the findings reported above. Thus, any changes in emotion-generative or cognitive control-related regions may simply reflect habituation or practice effects. To test this possibility, we conducted a supplementary experiment to repeatedly present stimuli for watching and GI, two conditions exhibiting little emotion regulation effect, for three times.

Accordingly, we conducted a 2 × 3 ×2 factorial experiment with the between factor self-regulation condition (watching and GI) and the within-factors type of pictures (neutral and disgust) and repetition times (1-3 times). One of our previous studies observed no significant habituation to repeatedly presented negative stimuli in emotional rating or brain potentials, suggesting that the humans ‘emotional reaction to negative stimuli are resistant to habituation (Long, at al., 2015). Also, the AREA (attend, react, explain, adapt) model of affective adaptation holds that the affective reactions to negative events would not decrease significantly until the negative events are fully understood (Wilson & Gilbert, 2008). However, the cues for effective explanation were unavailable during watching and GI. Based on these studies, we predict that repetition of emotional stimuli for watching or GI condition would not decrease one’s emotional responses to disgusting stimuli.

Results of three-factorial ANOVAs yielded neither significant interaction effects between repetition times and self-regulation conditions (F(2,76)=1.92, p=0.15, η^2^=0.05), nor the main effects of repetition times (F(2,76)=2.09, p=0.13, η^2^=0.05, **S-Figure 3**). There was a significant main effect of picture type (F(1,38)=294.98, p<0.001, η^2^=0.87). Bayesian analysis showed that the model including the valence factor has a largest BF_10_>100, providing strong evidence for accepting H1, whereas the model including the repetition times (0.051) or group (0.422) has a small BF_10_, providing moderate and anecdotal evidence for accepting H0, respectively.

Consistent with our prediction, these findings suggest that disgust experiences would not decrease as a result of emotional habituation or GI practice. Importantly, the findings of the present two experiments are consistent with the AREA (Attend, React, Explain and Adapt) model of affective adaptation (Wilson & Gilbert, 2008). In this model, Wilson and Gilbert (2008) proposed that people engage in the sequential process of attending, reacting, explaining, and ultimately adapting to affective events. The most novel part of AREA model is that explanation leads to affective adaptation. That is, affective reactions to negative events would not decrease significantly over time until individuals explain negative events successfully. RII, as a combination of cognitive reappraisal and implementation intentions, provided participants with effective explanations of disgust stimuli, whereas GI and watching conditions did not. Therefore, the decrease in subjective ratings and amygdala activity during RII compared to watching or GI in the main experiment can result exclusively from the regulatory effects of RII.


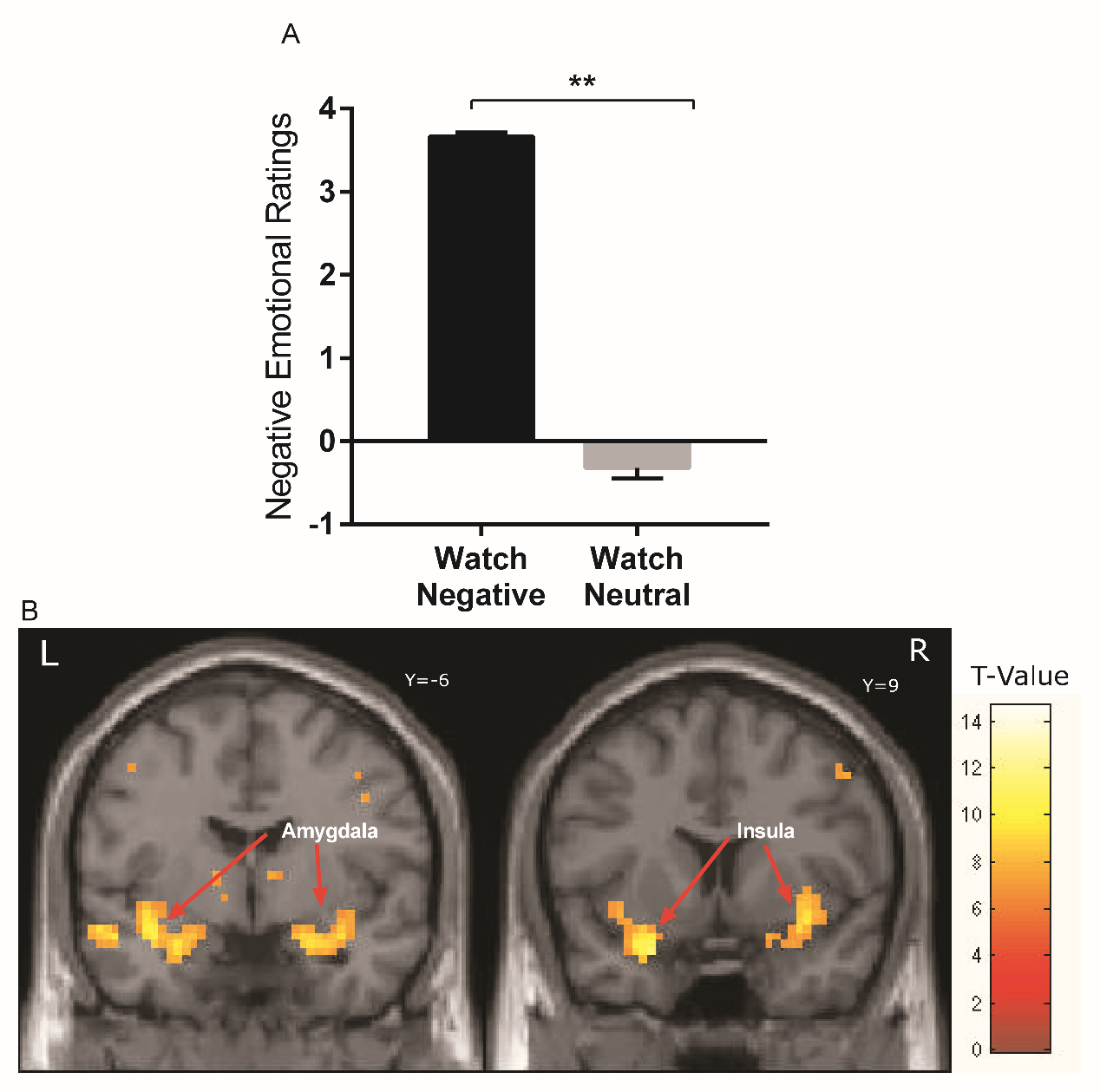


**S-Figure 1.** The contrast of watching-negative versus watching-neutral on behavioral and neural indices. (**A**) Negative emotional ratings during passively viewing of disgust pictures (watching condition). Error bars= SE. (**B**) From the one-sample t-test across all 26 participants for the contrast watching-negative versus watching-neutral. The display threshold was p=.01, FWE corrected and an extent of 10 voxels. ** means p≤0.01


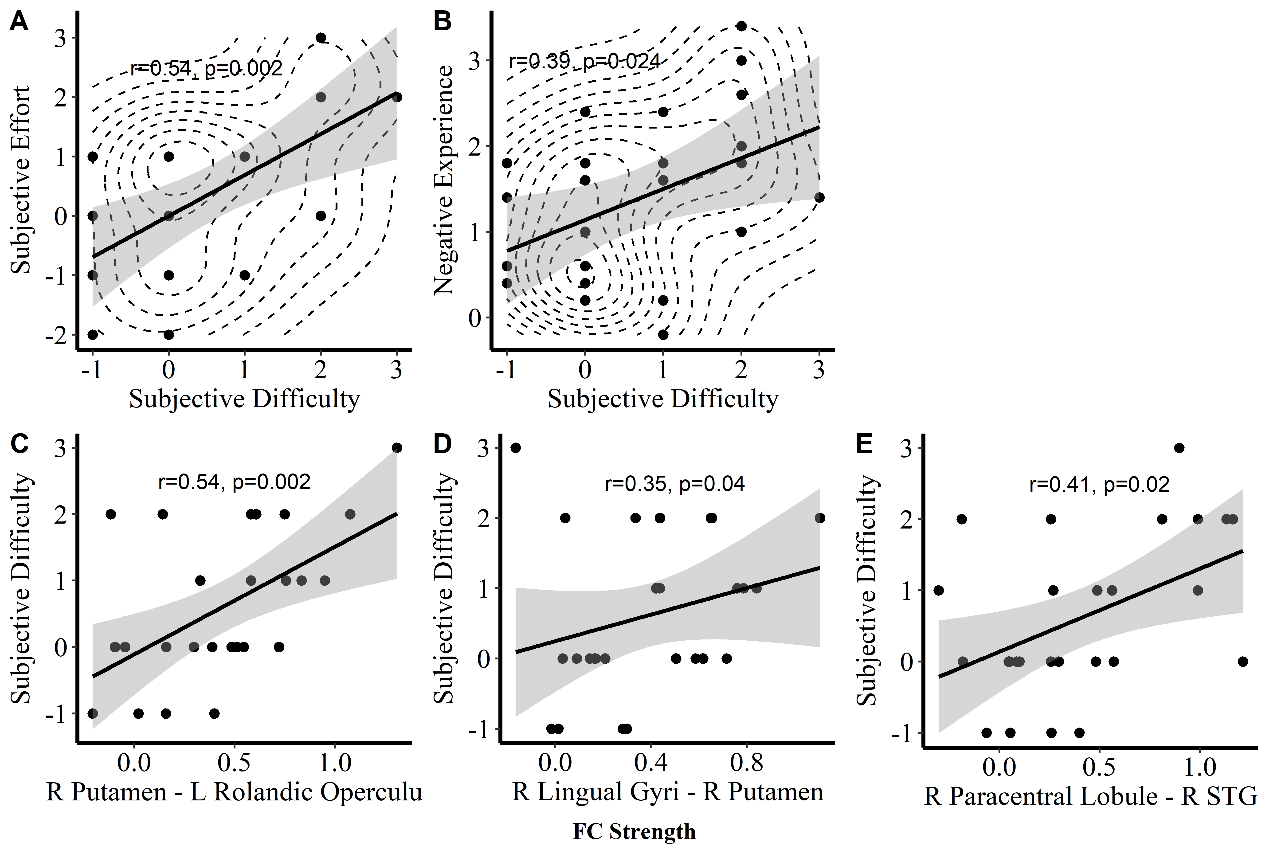
**S-Figure 2**. The correlation analysis showed that during GI relative to RII, subjective regulatory difficulty was related to greater subjective cognitive efforts (**A**) and negative experiences (**B**), and that the changes of FC intensity were related to greater subjective regulatory difficulty. Results of R Putamen - L Rolandic Operculum (**C**) survived a FDR of 0.05 correction for multiple comparisons, whereas R Lingual gyri – R Putamen (**D**) and R Paracentral Lobule - R STG (**E**) did not. Contour line density in **A** and **B** means the extent of point overlap.


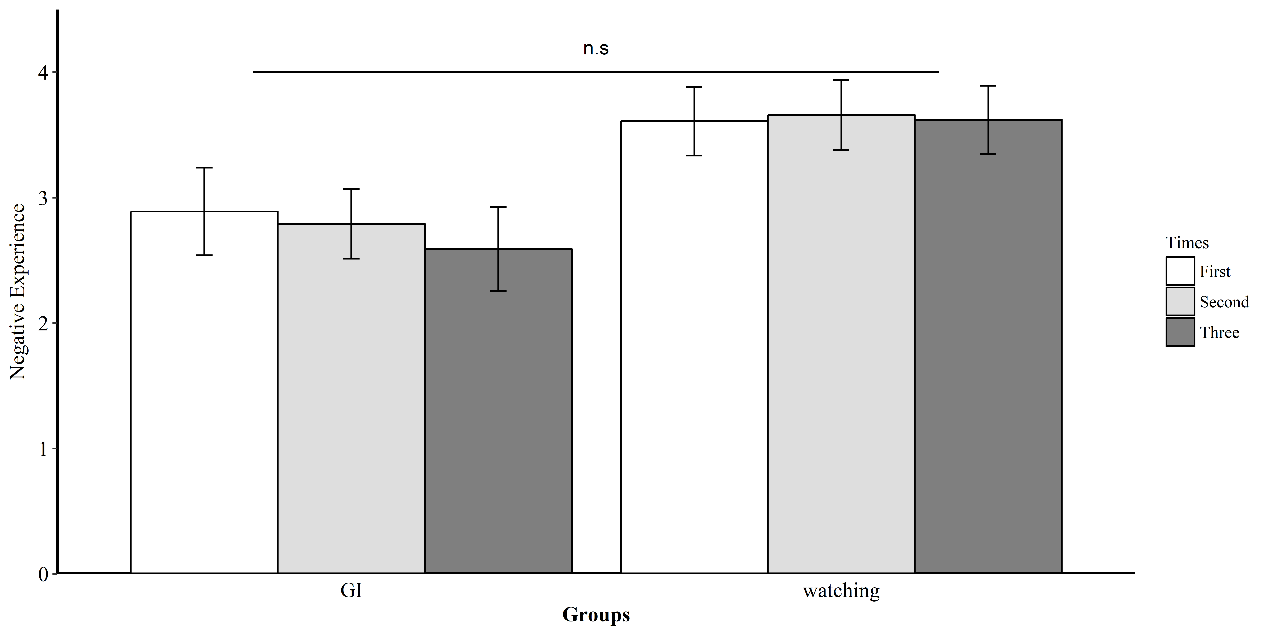


**S-Figure 3**. Mean subjective ratings of negative emotions for the watching and GI groups during three times. The negative affect during each condition was represented by the negative emotion rating minus the neutral emotion rating, and its higher values mean more negatively emotional experiences during the condition. Error bars=SEM, ns stands for not significant.

**S-Table 1.** Group Activations for Contrast Watching-negative Versus Watching-Neutral

| **Brain Regions** | **Brodmann** | **x** | **y** | **z** | **Voxels** | **t-Value** |
| --- | --- | --- | --- | --- | --- | --- |
|  |  |  |  |  |  |  |
| **Watching-negative>Watching-Neutral** |  |  |  |  |  |  |
| Frontal Lobes |  |  |  |  |  |  |
| L Anterior Cingulum | 32 | -12 | 42 | 6 | 94 | 11.46 |
| R Medial Superior Frontal | 10 | 3 | 63 | 18 | 576 | 10.57 |
| R Inferior Frontal Orbitalis | 47 | 39 | 33 | -18 | 37 | 9.69 |
| L Medial Superior Frontal | 10 | -12 | 63 | 18 | 24 | 9.59 |
| R Insula | 48 | 24 | 15 | -18 | 240 | 9.54 |
| L Middle Cingulum | 23 | -12 | -24 | 36 | 38 | 9.50 |
| R Inferior Frontal Triangle | 48 | 36 | 18 | 18 | 304 | 8.71 |
| L Insula | 48 | -36 | 6 | -6 | 67 | 8.43 |
| L Rectus | 11 | -6 | 39 | -18 | 91 | 5.52 |
| Temporal Lobes |  |  |  |  |  |  |
| R Middle Temporal Lobes | 37 | 48 | -72 | 3 | 948 | 14.63 |
| L Middle Temporal Lobes | 37 | -57 | -57 | 6 | 847 | 11.35 |
| R Fusiform | 19 | 27 | -57 | -12 | 364 | 11.16 |
| Parietal Lobes |  |  |  |  |  |  |
| L Postcentral | 3 | -33 | -39 | 54 | 108 | 8.28 |
| L Inferior Parietal | 3 | -45 | -24 | 39 | 334 | 7.46 |
| Occipital Lobes |  |  |  |  |  |  |
| R Middle Occipital | 37 | 48 | -72 | 3 | 142 | 14.63 |
| L Middle Occipital | 19 | -48 | -78 | 6 | 941 | 12.49 |
| R Lingual | 18 | 15 | -72 | -15 | 128 | 10.74 |
| L Lingual | 19 | -24 | -66 | -9 | 38 | 8.16 |
| Subcortical Regions |  |  |  |  |  |  |
| L Thalamus |  | -27 | -21 | -6 | 33 | 11.53 |
| R Hippocampus |  | 24 | -6 | -18 | 135 | 10.66 |
| R Amygdala |  | 30 | 0 | -21 | 27 | 10.46 |
| L Hippocampus |  | -24 | -9 | 15 | 273 | 10.35 |
| L Amygdala |  | -24 | -6 | -15 | 220 | 10.18 |
| **Watching-Neutral > Watching-negative** |  |  |  |  |  |  |
| No significant clusters of activation |  |  |  |  |  |  |

*Note.* All clusters reached a significance level of p=0.01 (FWE corrected). For each cluster, x, y, z, MNI coordinates; L, left; R, right.

**S-Table 2**. Results of 3-by-2 ANOVA of Functional Connectivity Analysis

| **FC** | | **Power’s Labels** | **F-value** |
| --- | --- | --- | --- |
|  |  |  |  |
| R Putamen - L Rolandic Operculum | | 41-53 | 25.27 |
| R Middle Occipital Gyri - R Middle Occipital Gyri | | 69-122 | 20.36 |
| R Inferior Temporal Gyri - L Middle Temporal Gyri | | 157-218 | 19.76 |
| R Lingual - R Putamen | | 121-204 | 18.58 |
| R Middle Occipital Gyri - L Inferior Temporal Gyri | | 151-225 | 18.41 |
| L vACC - R Supramarginal Gyri | | 100-178 | 16.76 |
| L Lingual Gyri - R Cerebellum 6 | | 66-143 | 16.71 |
| L vACC - L Insula | | 100-44 | 13.52 |
| R Inferior Parietal Lobule - R Superior Parietal Lobule | | 32-27 | 13.39 |
| R Postcentral Gyri - R Paracentral Lobule | | 27-209 | 13.31 |
| R Paracentral Lobule - R Superior Temporal Gyri | | 102-82 | 12.64 |
| L vACC - R Precuneus | | 221-035 | 11.72 |

*Note.* All connections reached a significance level of p<0.02, seed-based-FDR corrected. FC, Functional Connectivity. For each connection, L, left; R, right. Coordinates for each ROI can be found in Supplementary file_229_ROIs/Power 227 according to Power’s Labels.

**References**

Beck, A., Steer, R., & Brown, G. (1996). BDI-II, Beck depression inventory: manual: Psychological Corp. *San Antonio, TX*.

Bradley, M. M., & Lang, P. J. (1994). Measuring emotion: the Self-Assessment Manikin and the Semantic Differential. *Journal of Behavior Therapy and Experimental Psychiatry, 25*(0005-7916 (Print)), 49-59.

Spielberger, C. D. (1970). STAI manual for the state-trait anxiety inventory. *Self-Evaluation Questionnaire*, 1-24.
